# Bixin and Norbixin Have Opposite Effects on Glycemia, Lipidemia, and Oxidative Stress in Streptozotocin-Induced Diabetic Rats

**DOI:** 10.1155/2014/839095

**Published:** 2014-01-30

**Authors:** Miguel Roehrs, Cassieli Gehlen Figueiredo, Mariane Magalhães Zanchi, Guilherme Vargas Bochi, Rafael Noal Moresco, Andréia Quatrin, Sabrina Somacal, Lisiane Conte, Tatiana Emanuelli

**Affiliations:** ^1^Graduate Program on Pharmacology, Center of Health Sciences, Federal University of Santa Maria, 97105-900 Santa Maria, RS, Brazil; ^2^Integrated Center for Laboratory Analysis Development (NIDAL), Department of Alimentary Technology and Science, Center of Rural Sciences, Federal University of Santa Maria, 97105-900 Santa Maria, RS, Brazil; ^3^Department of Clinical and Toxicological Analysis, Center of Health Sciences, Federal University of Santa Maria, 97105-900 Santa Maria, RS, Brazil

## Abstract

The present study investigated the effects of oral administration of annatto carotenoids (bixin (BIX) and norbixin (NBIX)) on glucose levels, lipid profiles, and oxidative stress parameters in streptozotocin (STZ)-induced diabetic rats. Animals were treated for 30 days in the following groups: nondiabetic control, diabetic vehicle, diabetic 10 mg/kg BIX, diabetic 100 mg/kg BIX, diabetic 10 mg/kg NBIX, diabetic 100 mg/kg NBIX, diabetic metformin, and diabetic insulin. Blood glucose, LDL cholesterol, and triglyceride levels were reduced in the diabetic rats treated with BIX. BIX treatment prevented protein oxidation and nitric oxide production and restored superoxide dismutase activity. NBIX treatment did not change most parameters assessed, and at the highest dose, it increased LDL cholesterol and triglycerides levels and showed prooxidant action (increased protein oxidation and nitric oxide levels). These findings suggested that BIX could have an antihyperglycemic effect, improve lipid profiles, and protect against damage induced by oxidative stress in the diabetic state. Because NBIX is a water-soluble analog of BIX, we propose that lipophilicity is crucial for the protective effect of annatto carotenoids against streptozotocin-induced diabetes.

## 1. Introduction

Diabetes mellitus (DM) is a chronic metabolic disorder that continues to be a major health problem worldwide. It affects approximately 6% of the population worldwide or 371 million individuals [1]. DM is characterized by hyperglycemia and alterations in carbohydrate, fat, and protein metabolism [2]; impairments in antioxidant enzymes [3]; high oxidative stress-induced damage to pancreatic *β* cells [4]. This disorder is caused either by a deficiency in the production of insulin or by a hyperinsulinemic condition that is accompanied by tissue resistance to insulin [2].

In addition, plasma triglycerides, low-density lipoprotein (LDL), and total cholesterol levels are usually elevated in diabetes, whereas the levels of high-density lipoproteins (HDL) are reduced [5]. The dyslipidemia associated with the hyperglycemia and changes in the sensitivity or reactivity of the vascular smooth muscle to neurotransmitters and circulating hormones plays a role in the accelerated development of atherosclerotic vascular disease, which is a major long-term complication of diabetes in humans [6].

Hyperglycemia has been hypothesized to contribute to oxidative stress either through the direct generation of ROS or by altering the redox balance [7]. This is thought to occur via several well-studied mechanisms, including increased polyol pathway flux, increased intracellular formation of advanced glycation end products (AGEs), activation of protein kinase C, or overproduction of superoxide by the mitochondrial electron transport chain [7, 8]. The polyol pathway leads to a NADPH-dependent reduction of glucose to sorbitol via aldose reductase. Under hyperglycemic conditions, the glucose flux through the polyol pathway is increased, which decreases the levels of NADPH and contributes greatly to an overall redox imbalance in the cell that leads to oxidative stress [8]. In addition, hyperglycemia increases the production of AGEs, which are formed through the covalent binding of aldehyde or ketone groups from reducing sugars to free amino groups of proteins, creating a Schiff base. Glucose alone can also undergo autoxidation to form reactive carbonyl intermediates, of which glyoxal and methylglyoxal are the two main intermediates. These reactive carbonyl intermediates then complete a complex series of chemical rearrangements to yield irreversible AGE structures [9]. Another important source of ROS is the overproduction of superoxide by the mitochondrial electron transport chain. This overproduction occurs under hyperglycemic conditions because the number of substrates entering the Krebs cycle is greatly increased, and consequently, the number of reducing equivalents donating electrons to the mitochondrial electron transport chain is also increased [8].

**Figure 1:**
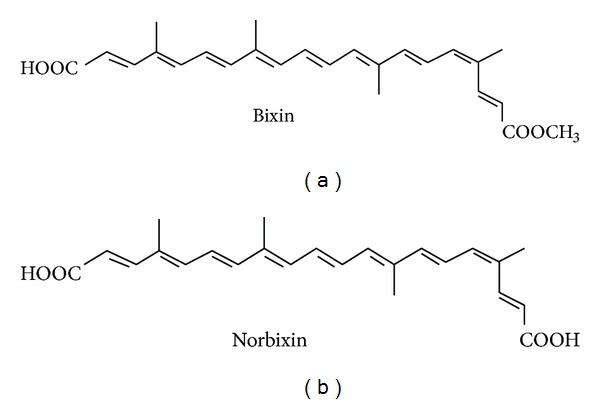
Chemical structure of annatto seed carotenoids.

Therefore, antioxidant compounds such as tea catechins, resveratrol, and garlic acid have been demonstrated to have benefits in the prevention and treatment of diabetic complications caused by oxidative stress [10–12]. The seeds of *Bixa orellana* L., a native shrub from tropical America, are a rich source of antioxidant carotenoid pigments that are largely used by the food coloring industry. This pigment, which is known as annatto (E160b), contains approximately 80% bixin (BIX; Figure 1(a)), an unusual lipid-soluble carotenoid that has a free carboxyl and an esterified carboxyl end group [13]. The hydrolytic removal of the methyl ester group from BIX by saponification generates norbixin (NBIX; Figure 1(b)), a water-soluble carotenoid also found in annatto preparations but at lower amounts than BIX [14]. These annatto carotenoids have been shown to have important antioxidant effects [15].

In addition, annatto extract can cause hypoglycemic episodes in normoglycemic dogs that are mediated by increased insulin plasma levels and possibly by increased affinity of insulin for its receptors [16]. However, this effect is dependent on the solvent used to obtain the annatto extract because oily extracts have a hypoglycemic effect, whereas ethanolic extracts have a hyperglycemic effect in normoglycemic dogs [16, 17]. In addition, NBIX has been shown to have opposing effects in normoglycemic rats and mice: it has a hyperglycemic effect in rats but a hypoglycemic effect in mice [18]. In fact, a study using adipocyte cell culture showed that BIX regulated the mRNA expression of PPAR-*γ* target genes that are involved in adipogenesis and improved insulin sensitivity [19]. Furthermore, it has been shown that both BIX and NBIX are agonists of the PPAR-*α* receptor, which regulates the expression of genes involved in fatty acid oxidation and carbohydrate metabolism [20].

These previous studies in cell culture and normoglycemic healthy animals have provided important evidence that annatto carotenoids may have positive effects on carbohydrate metabolism. Thus, studies in animal models of diabetes could help to determine the pharmacological potential of these carotenoids to control risk factors for diabetes complications. Given that oxidative stress has been associated with the pathogenesis of various diabetic complications [3, 5] and given the antioxidant effects of annatto in other biological models, we hypothesized that the annatto carotenoids could also directly counteract the oxidative stress associated with diabetes in addition to acting on the glucose metabolism. Thus, the present study investigated whether BIX and NBIX could prevent the hyperglycemia, dyslipidemia, and oxidative stress associated with streptozotocin (STZ)-induced diabetic rats, which is a well-characterized animal model of diabetes [21]. The effects of these annatto carotenoids were compared to the effects of standard hypoglycemic drugs used in the treatment of diabetes.

## 2. Materials and Methods

### 2.1. Chemicals and Solutions

STZ, DL-dithiothreitol (DTT), glutathione, 5,5′-dithiobis(2)-nitrobenzoic acid (DTNB), sodium nitrate, vanadium (III) chloride, sulphanilamide, N-(1-naphthyl)ethylenediamine, glutathione reductase, epinephrine hydrochloride, *β*-nicotinamide adenine dinucleotide 2′-phosphate reduced tetrasodium salt hydrate, and L-glutathione oxidized disodium salt were obtained from Sigma-Aldrich (St. Louis, MO, USA). The orthophosphoric acid, hydrochloric acid, potassium iodide, acetic acid, and sodium hydroxide were purchased from Vetec (Brazil). Metformin (99%) was from Valdequimica (São Paulo, SP, Brazil) and the NPH insulin (100 IU/mL) used was commercially purchased (Lilly, Indianapolis, IN, USA). All other reagents used in the experiments were of analytical grade and of the highest purity.

BIX oily solution (10%) and NBIX aqueous solution (10%) were donated by Christian Hansen (Brazil). BIX and NBIX solutions at 5 and 50 mg/mL were prepared by dilution in distilled water containing 10% Tween 80 and 0.5% ethanol. The vehicle aqueous solution contained 10% Tween 80 and 0.5% ethanol. Metformin was dissolved in water at 50 mg/mL. The solutions were prepared three times a week and stored in amber bottle at 2 to 8°C until use.

### 2.2. Animals and Streptozotocin-Induced Diabetes

Adult male Wistar rats (70–90 days; 200–350 g) from the Central Animal House of the Federal University of Santa Maria were housed under controlled temperature (23 ± 1°C) and humidity (56%) on a 12-hour light-dark cycle with free access to food (Supralab, São Leopoldo, RS, Brazil) and water. Before the beginning of the experiments, animals were adapted in cages for 20 days. All the experimental procedures were carried out in accordance with international guidelines for care and use of laboratory animals (Council of European Communities, 1986) and were approved by the Committee on Care and Use of Experimental Animal Resources of the Federal University of Santa Maria (protocol no.: 089/2011). Diabetes was induced by a single i.p. injection of STZ (1 mL/kg b.w., 60 mg/kg b.w.) dissolved in sodium-citrate buffer (0.05 M, pH 4.5) [21]. The control group received vehicle (sodium-citrate buffer, 1 mL/kg b.w.). In order to reduce death due to hypoglycemic shock, STZ-treated rats received glucose (20%, 2 mL/kg b.w.) by gavage 6 hours after diabetes induction. In addition, during the first 24 h after STZ administration a 5% glucose solution was offered to the animals instead of water. Then, the animals were kept for 15 days with free access to food and water before glycemia evaluation [21]. Blood glucose levels were measured with an automatic analyzer donated by Roche of Brazil (Advantage, Boehringer Mannheim, Indianapolis, IN, USA). Only animals with fasting glycemia over 200 mg/dL were considered diabetic.

### 2.3. Experimental Design

The animals were randomly divided into eight groups (6 rats per group): nondiabetic control + vehicle, diabetic + vehicle, diabetic + 10 mg/kg BIX, diabetic + 100 mg/kg BIX, diabetic + 10 mg/kg NBIX, diabetic + 100 mg/kg NBIX, diabetic + 100 mg/kg metformin, and diabetic + insulin (4 IU). Vehicle, BIX, NBIX, and metformin were given by gavage (up to 2 mL/kg b.w.) as a daily administration for 30 days. The insulin dose was fractionated (2 IU doses) and administered intradermically twice a day for 30 days. BIX and NBIX doses were chosen based on a previous study [18]. During all the experiments the animals had free access to food and water. The animals were weighed before the diabetes induction and once a week along the experimental period.

After the 30 days of treatment, animals were fasted overnight and anesthetized with xylazine (10 mg/kg body weight) and ketamine (75 mg/kg body weight) and blood samples were collected by cardiac puncture into heparinized tubes to measure the activity of antioxidant enzymes. Another blood sample was collected without anticoagulant to obtain serum for the biochemical analyses and to assess the products of advanced protein oxidation (AOPP) and nitric oxide (NOx) levels.

### 2.4. Biochemical Analyses

The serum concentrations of glucose, total cholesterol, triglycerides, and HDL were determined using colorimetric kits (Doles, Goiania, GO, Brazil). The LDL cholesterol level was calculated according to Friedewald et al. [22]. Fructosamine was measured using a kinetic kit assay (Gold Analisa, Belo Horizonte, MG, Brazil). Insulin was measured by radioimmunoassay (Immunotech, Beckman Coulter Company, Marseille, France).

### 2.5. Biomarkers of Oxidative Stress

Serum NOx levels were assessed as nitrite/nitrate content measured on Cobas MIRA as previously described [23]. The oxidation of proteins was evaluated by determining the products of advanced protein oxidation (AOPP) [24]. Glutathione peroxidase (GPx) activity was determined by spectrophotometry using glutathione reductase and NADPH [25]. Catalase (CAT) activity was assessed by spectrophotometry, using hydrogen peroxide as previously described [26]. Superoxide dismutase (SOD) activity was determined by spectrophotometry using epinephrine [27]. To measure thioredoxin reductase (TrxR) activity, total blood was hemolyzed with four volumes of cold Milli-Q water and centrifuged at 9,000 ×g for 15 min at 4°C. The supernatant was then diluted and used for an enzyme assay based on the reduction of DTNB to 5′-thionitrobenzoic acid (TNB) at 412 nm [28]. Glutathione reductase (GR) activity was determined using oxidized glutathione and NADPH. The method is based on the oxidation of NADPH, which is indicated by a decrease in absorbance at 340 nm [29].

### 2.6. Statistical Analysis

The results were expressed as the mean ± SEM. Data were analyzed for statistical significance by one-way ANOVA followed by Duncan's test using the Statistica 7.0 and differences were considered significant at *P* < 0.05.

## 3. Results

### 3.1. Body Weight, Blood Glucose, Fructosamine, and Insulin Levels

**Figure 2:**
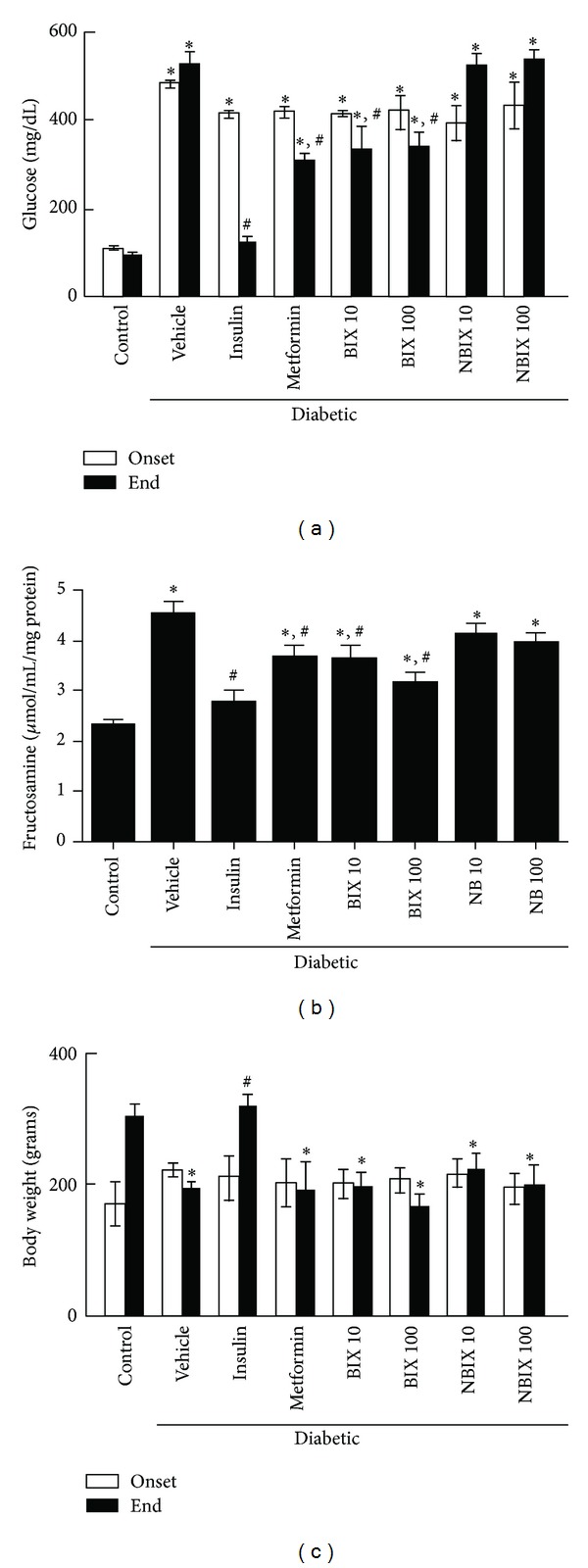
Blood glucose (a) and fructosamine levels (b) and body weight (c) of streptozotocin-induced diabetic rats. Blood glucose levels and body weight were assessed at the onset and end of treatment with vehicle, bixin, norbixin, insulin, or metformin. Fructosamine levels were assessed at the end of the treatment. Data are expressed as means ± SEM (*n* = 6 per group). ANOVA followed by Duncan's test: *P < 0.05 versus the respective nondiabetic control; ^#^ *P* < 0.05 versus the respective diabetic vehicle. BIX 10: 10 mg/kg bixin; BIX 100: 100 mg/kg bixin; NBIX 10: 10 mg/kg norbixin; NBIX 100: 100 mg/kg norbixin.

STZ-induced diabetes increased blood glucose levels 4-fold compared to the nondiabetic control rats (*P* < 0.05; onset levels in Figure 2(a)). Diabetic rats treated for 30 days with BIX (10 or 100 mg/kg) had a reduction in blood glucose levels compared to the diabetic vehicle group (*P* < 0.05; end levels in Figure 2(a)). This reduction was similar to that observed after treatment with metformin, but it was lower than that observed after treatment with insulin, which reduced blood glucose levels to a value similar to the nondiabetic control group (*P* < 0.05; end levels in Figure 2(a)). In contrast, the diabetic rats treated with NBIX had no change in blood glucose levels compared to the diabetic vehicle group (end levels in Figure 2(a)).

Fructosamine levels increased 2-fold in the diabetic animals compared to the nondiabetic control rats (*P* < 0.05; Figure 2(b)). The treatment with BIX (10 or 100 mg/kg) reduced fructosamine levels of diabetic rats and this effect was similar to that of metformin (*P* < 0.05; Figure 2(b)). Insulin was the most effective treatment to reduce fructosamine levels of diabetic rats (*P* < 0.05), whereas NBIX had no effect (Figure 2(b)). Fasting blood insulin levels were not different among groups at the end of the treatment (data not show).

No significant difference was observed in the body weight among groups at the onset of the treatment (Figure 2(c)). However, after 30 days diabetic rats treated with vehicle, BIX, NBIX, or metformin had lower body weight than diabetic rats treated with insulin and nondiabetic control animals (Figure 2(c); *P* < 0.05). This occurred because only these two latter groups gained weight during the experimental period (53 and 79%, resp.), whereas the other groups had no body weight change.

### 3.2. Lipid Profile

**Figure 3:**
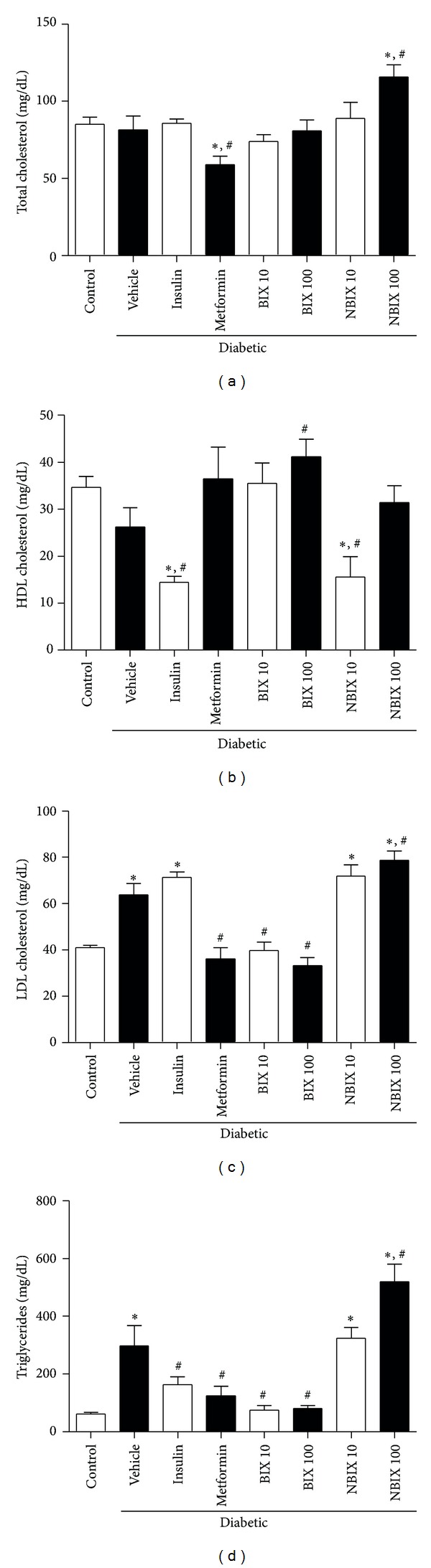
Lipid profile of streptozotocin-induced diabetic rats: total cholesterol (a), HDL cholesterol (b), LDL cholesterol (c), and triglycerides (d). Data are expressed as means ± SEM (*n* = 6 per group). ANOVA followed by Duncan's test: *P < 0.05 versus nondiabetic control; ^#^ *P* < 0.05 versus diabetic vehicle. BIX 10: 10 mg/kg bixin; BIX 100: 100 mg/kg bixin; NBIX 10: 10 mg/kg norbixin; NBIX 100: 100 mg/kg norbixin.

Total and HDL cholesterol levels were similar between nondiabetic and diabetic rats (Figures 3(a) and 3(b)). However, treatment with metformin decreased the total cholesterol levels of diabetic rats, whereas 100 mg/kg NBIX increased these levels compared to the diabetic vehicle group (*P* < 0.05; Figure 3(a)). Treatment with insulin or 10 mg/kg NBIX decreased HDL cholesterol levels compared to the nondiabetic and diabetic vehicle rats, whereas treatment with 100 mg/kg BIX increased HDL levels compared to the diabetic vehicle rats (Figure 3(b); *P* < 0.05).

On the other hand, the LDL cholesterol and triglycerides levels were significantly increased in the diabetic vehicle group compared to nondiabetic control rats (*P* < 0.05; Figures 3(c) and 3(d)). Treatment with BIX (10 or 100 mg/kg) or metformin reduced LDL cholesterol levels of diabetic rats to values similar to those of the nondiabetic control rats (Figure 3(c); *P* < 0.05). However, insulin or 10 mg/kg NBIX did not change LDL levels compared to the diabetic vehicle group, whereas 100 mg/kg NBIX increased these levels (*P* < 0.05; Figure 3(c)). Treatment with BIX (10 or 100 mg/kg) reduced triglycerides levels (*P* < 0.05) to values similar to those of the nondiabetic control group and this effect was similar to that observed after insulin or metformin treatment (Figure 3(d)). NBIX at 10 mg/kg did not change triglycerides levels but at 100 mg/kg it increased these levels when compared to the diabetic vehicle rats (Figure 3(d); *P* < 0.05).

### 3.3. Biomarkers of Oxidative Stress

**Figure 4:**
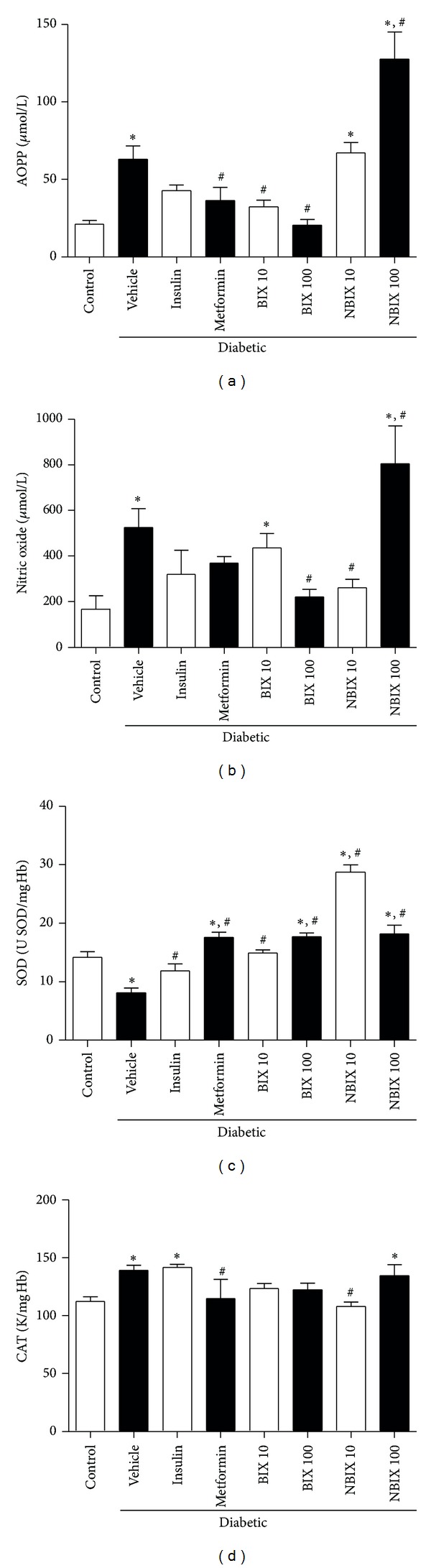
Oxidative stress parameters of streptozotocin-induced diabetic rats: AOPP levels (a), nitric oxide levels (b), superoxide dismutase (SOD) (c), and catalase activity (CAT) activity (d). Data are expressed as mean ± SEM (*n* = 6 per group). ANOVA followed by Duncan's test: *P < 0.05 versus nondiabetic control; ^#^ *P* < 0.05 versus diabetic vehicle. BIX 10: 10 mg/kg bixin; BIX 100: 100 mg/kg bixin; NBIX 10: 10 mg/kg norbixin; NBIX 100: 100 mg/kg norbixin.

The oxidation of proteins, as assessed by AOPP, was increased in diabetic vehicle rats compared to the nondiabetic control group (Figure 4(a); *P* < 0.05). Treatment with BIX up to 100 mg/kg decreased the AOPP levels of diabetic rats, similar to that observed with metformin (Figure 4(a); *P* < 0.05). The diabetic rats treated with insulin also had no increase in AOPP levels compared to the nondiabetic control group, albeit its AOPP level was not different from the diabetic vehicle group (*P* < 0.05; Figure 4(a)). On the other hand, NBIX treatment (100 mg/kg) increased protein oxidation compared to the diabetic vehicle rats (Figure 4(a); *P* < 0.05).

The NOx serum levels were increased in the diabetic vehicle rats compared to the nondiabetic control (*P* < 0.05; Figure 4(b)). This increase was not observed after treatment with insulin, metformin, 100 mg/kg BIX, or 10 mg/kg NBIX (Figure 4(b)). In fact, treatments with 100 mg/kg BIX or 10 mg/kg NBIX were more effective than insulin or metformin to prevent this increase in NOx levels, because they caused a significant decrease of these levels compared to the diabetic vehicle group (*P* < 0.05; Figure 4(b)). However, treatment with 100 mg/kg NBIX increased NOx levels of diabetic rats compared to the diabetic vehicle group (*P* < 0.05; Figure 4(b)).

Superoxide dismutase activity was decreased in the diabetic vehicle rats compared to the nondiabetic control group (Figure 4(c); *P* < 0.05), but this decrease was recovered in all diabetic treated rats (*P* < 0.05; Figure 4(c)). In fact, the treatment with metformin, 100 mg/kg BIX, or NBIX (10 or 100 mg/kg) not only recovered the decrease of superoxide dismutase activity caused by diabetes, but also increased superoxide dismutase activity compared to the nondiabetic control (up to 2-fold for 10 mg/kg NBIX) (Figure 4(c)). Catalase activity was increased in the diabetic vehicle rats compared to the nondiabetic control group (Figure 4(d); *P* < 0.05), but this change was not observed in the diabetic rats treated with BIX (10 or 100 mg/kg), NBIX (10 mg/kg), or metformin (Figure 4(d)), indicating a protective effect of these treatments.

The activity of glutathione peroxidase was not altered by diabetes (Table 1). However, the treatment with insulin or 100 mg/kg NBIX increased glutathione peroxidase activity compared to the nondiabetic control and to the diabetic vehicle group (*P* < 0.05; Table 1). The activities of glutathione reductase and thioredoxin reductase were not altered by diabetes, but diabetic rats treated with 100 mg/kg BIX had higher enzyme activities than the diabetic vehicle group or the nondiabetic control group (*P* < 0.05; Table 1). In contrast, diabetic rats treated with 10 mg/kg NBIX had a decrease in thioredoxin reductase activity compared to the diabetic vehicle group and to the nondiabetic control group (*P* < 0.05; Table 1).

## 4. Discussion

STZ-induced diabetes models are the most used animal models of diabetes because they reproduce in full the pathogenesis of this disease. STZ is accumulated via GLUT2 transporter in the pancreatic *β* cells, which are then massively destroyed [30]. Moreover, models of insulin-deficient diabetes result in increased GLUT4 phosphorylation, which may render GLUT4 less sensitive to acute regulation by insulin [31]. In our study this model was effectively reproduced and all diabetic groups showed 4-fold increase in blood glucose levels.

In this study, we found that treatment with BIX (10 or 100 mg/kg) for 30 days reduced blood glucose as well as fructosamine levels of diabetic rats. The antihyperglycemic effect of BIX seems to have occurred soon after the start of the treatment, because the decreased level of fructosamine indicates that protein glycation was reduced in the last three weeks [32]. This antihyperglycemic effect could be explained by the activation of PPAR-*γ* receptors by BIX, a mechanism that was demonstrated *in vitro* in adipocytes cultures [33]. In fact, agonists of this receptor, like the thiazolidinediones, are known to have anti-diabetic effects that are related to the stimulation of adipocyte differentiation leading to increased number of insulin sensitive small adipocytes [33, 34]. The antihyperglycemic effect of BIX was similar to that achieved by the oral anti-diabetic metformin, which is mainly used to treat noninsulin-dependent diabetes in humans. Metformin reduces plasma glucose levels by enhancing tissue sensitivity to insulin and it is not effective in the absence of insulin. Thus, we cannot rule out that the antihyperglycemic effect of BIX could be related to changes in GLUT4 expression or due to GLUT4 dephosphorylation and attenuation of hepatic PEPCK gene expression as previously demonstrated for metformin [35].

In contrast to BIX, the treatment with NBIX did not reduce fasting blood glucose levels. The difference between these structurally similar annatto carotenoids could be related to their different polarity. BIX has a lipophilic character, whereas NBIX has a hydrophilic character. Our results suggest that NBIX had no effect in the PPAR-*α* or *γ* receptors in this diabetes model. In fact, the cell culture studies on the activation of PPAR receptors by annatto carotenoids showed a lower potency for NBIX compared to BIX [17, 20].

In addition to the antihyperglycemic effect, BIX (10 and 100 mg/kg) completely prevented the increase in serum triglycerides and LDL cholesterol levels that occurred in diabetic rats. Moreover, the highest dose of BIX also increased the HDL levels of diabetic rats. This effect is similar to that recently demonstrated for an aqueous extract of annatto in a model of hypercholesterolemia in rats [36]. Interestingly, our results demonstrated that BIX was more effective than currently used anti-diabetic drugs to improve the lipid profile in diabetes. Metformin had no beneficial effect on HDL levels, whereas insulin did not prevent changes in LDL levels and it indeed decreased the HDL levels of diabetic rats. In contrast to BIX, the treatment with NBIX did not protect against changes in the lipid levels and at the highest dose it even increased total cholesterol, LDL, and triglyceride levels compared to the diabetic vehicle rats. The beneficial effect of BIX on the lipid profile may be related to the activation of PPAR-*α* receptor, which increases the expression of genes involved in fatty acid oxidation, fatty acid uptake, and energy consumption in the liver as recently demonstrated in obese KK-Ay mice [20]. This receptor is expressed primarily in tissues with high rates of fatty acid oxidation and peroxisomal metabolism [37] and is activated by several compounds of the isoprenoid family, such as BIX [38, 39]. The effect of NBIX on the PPAR-*α* receptor was only preliminarily investigated in cell culture assays, where NBIX had a lower potency than BIX to activate the PPAR-*α* in a GAL4/PPAR-*α* chimera system [20]. Thus, our results suggest that NBIX is unlikely to activate the PPAR-*α* receptor in the STZ model of diabetes in rats, which could be related to its lower hydrophobicity compared to BIX [40].

The exposure to high glucose levels may increase the generation of ROS through the nonenzymatic glycation of proteins and glucose auto-oxidation, which leads to oxidative stress and structural and functional tissue damage [41, 42]. The immunological effector molecules in this process are cytokines, namely, interleukin-1 [43], which activates the expression of inducible nitric oxide synthase. High local NOx concentrations induce oxidative stress followed by the destruction of pancreatic *β* cells and subsequent development of insulin-dependent diabetes mellitus. Moreover, several studies have shown that a single dose of STZ increases the levels of NOx, being responsible for the damage to *β* cells that leads to diabetes in experimental models [44]. In our study, the diabetic animals had increased NOx levels compared to the nondiabetic control group and this increase was prevented by treatment with the highest dose of BIX and the lowest dose of NBIX. In contrast, treatment with the highest dose of NBIX increased NOx levels to values even higher than the diabetic vehicle group.

AOPP is a suitable biochemical marker for measuring short-term changes in oxidative stress because it assesses the products of plasma protein oxidation, especially albumin oxidation. This marker is increased in inflammatory conditions such as diabetes, atherosclerosis, and renal failure [45]. In the present study, BIX (10 and 100 mg/kg) prevented the increase in AOPP levels caused by the diabetes and this protective effect was similar to that of the antihyperglycemic drugs metformin and insulin. In contrast, NBIX had no protective effect and at the highest dose it further increased AOPP levels of diabetic rats, indicating a prooxidant effect of this carotenoid.

Antioxidant enzymes are the first line of defense against reactive species and oxidative stress [46]. In the present study, diabetic rats had slightly increased catalase activity, which could be interpreted as a defense response against oxidative stress. Despite this response, diabetic rats had increased protein oxidation (AOPP levels). Thus, protein oxidation in diabetic rats could be related to the overproduction of NOx associated with the lower superoxide dismutase activity. Excessive NOx concentrations may react with superoxide anion radical (O_2_^•−^) to form peroxynitrite anion (ONOO^−^), which is a highly reactive oxidizing agent [47]. Treatment with BIX increased superoxide dismutase activity, which probably reduced superoxide anion levels. In addition, it also reduced NOx levels and protein oxidation, which is in agreement with the hypothesis that protein oxidation in diabetic rats was mainly mediated by peroxynitrite. Moreover, the increase in glutathione reductase and thioredoxin reductase activities caused by the treatment with 100 mg/kg BIX could also contribute to its protective effect against oxidative stress in this model of diabetes. NBIX 10 mg/kg decreased NOx levels and increased superoxide dismutase activity of diabetic rats, but it failed to protect against protein oxidation. In fact, NBIX 10 mg/kg increased SOD activity to levels much higher than control. This increased activity of superoxide dismutase along with the reduction of catalase activity leads to the accumulation of H_2_O_2_, because it is actively produced by superoxide dismutase but not efficiently removed by catalase. This imbalance in the removal of oxidative species was previously reported in various oxidative conditions including alloxan-induced diabetes [48] and is likely contributing to the oxidation of proteins in our study. The treatment with 100 mg/kg NBIX had a marked prooxidant effect by increasing 2-fold the protein oxidation of diabetic rats, which was probably related to the greater increase in NOx levels caused by this treatment.

Thus, BIX also showed antioxidant activity in the STZ-induced model of diabetes, in addition to the antihyperglycemic and antilipidemic effects. This finding is in agreement with the effectiveness of BIX and BIX-enriched annatto extracts to scavenge for both nitrogen and oxygen reactive species *in vitro*, which have been attributed to the electron transfer due to the presence of many conjugated double bonds [49]. Although they had better scavenging capacity for nonradical species (H_2_O_2_, HOCl, ^1^O_2_, and ONOO^−^) than for radical species (O_2_^•−^ and ^•^NO), we showed that BIX did decrease NOx levels in STZ-induced diabetes. In addition to the direct scavenging of reactive species, BIX may also enhance the antioxidant defenses, as demonstrated in the present study for superoxide dismutase, glutathione reductase, and thioredoxin reductase activities. The increased activity of these enzymes may be a result of an increased gene expression, since an annatto extract enriched in BIX was recently shown to prevent the decrease in the activity and expression of superoxide dismutase in neutrophils from alloxan-induced diabetic rats [50].

## 5. Conclusion

BIX, at doses that show no toxic effects [51], was as effective as metformin to decrease blood glucose levels and more effective than metformin and insulin to improve the dyslipidemia in STZ-induced diabetes. Moreover, BIX also prevented the oxidative stress in diabetic rats. Thus, BIX seems to be a promising drug for the diabetes therapy, which may be important considering the known side effects of drugs currently used in human therapy of diabetes [52]. In contrast, the treatment with NBIX, the hydrosoluble analog of BIX, was ineffective to protect against the hyperglycemia and dyslipidemia in STZ-induced diabetes. Furthermore, the highest dose of NBIX increased dyslipidemia and oxidative stress in the STZ-induced diabetes model. Thus, our results suggest that lipophilicity is crucial for the protective effect of annatto carotenoids against STZ-induced diabetes.

## Figures and Tables

**Table 1:** Effect of bixin and norbixin treatment on oxidative stress parameters in the blood of streptozotocin-induced diabetic rats.

Groups	GPx (nmol NADPH/min/g Hb)	GR (nmol NADPH/min/mg Hb)	TrxR (nmol DTNB reduced/min/mg Hb)
Nondiabetic control	26.3 ± 5.9	20.6 ± 1.2	155.5 ± 8.2
Diabetic vehicle	25.0 ± 1.6	32.0 ± 0.7	138.0 ± 16.5
Diabetic + insulin	49.6 ± 9.2^∗,#^	18.2 ± 2.9	172.7 ± 21.3
Diabetic + metformin	29.8 ± 8.3	23.9 ± 11.1	142.1 ± 11.4
Diabetic + bixin (10 mg/kg)	11.4 ± 3.7	19.2 ± 3.9	149.8 ± 6.5
Diabetic + bixin (100 mg/kg)	19.7 ± 6.5	61.8 ± 10.9^∗,#^	264.3 ± 27.0^∗,#^
Diabetic + norbixin (10 mg/kg)	26.0 ± 9.5	18.0 ± 1.2	96.3 ± 11.2^∗,#^
Diabetic + norbixin (100 mg/kg)	62.2 ± 3.2^∗,#^	17.2 ± 2.3	150.9 ± 12.7

Data are expressed as means ± SEM (*n* = 6  per group, except for the metformin and insulin groups that had 5 animals). ANOVA followed by Duncan's test: *P < 0.05 versus nondiabetic control; ^#^ *P* < 0.05 versus diabetic vehicle.
